# GC-MS Profile and Enhancement of Antibiotic Activity by the Essential Oil of *Ocotea odorífera* and Safrole: Inhibition of *Staphylococcus aureus* Efflux Pumps

**DOI:** 10.3390/antibiotics9050247

**Published:** 2020-05-12

**Authors:** Ray S. Almeida, Priscilla R. Freitas, Ana Carolina J. Araújo, Irwin R. Alencar Menezes, Eduardo L. Santos, Saulo R. Tintino, Talysson F. Moura, Jaime R. Filho, Vitória A. Ferreira, Ana Cristina A. Silva, Luiz E. Silva, Wanderlei do Amaral, Cícero Deschamps, Marcello Iriti, Henrique D. Melo Coutinho

**Affiliations:** 1 Department of Biological Chemistry, Regional University of Cariri-URCA, Crato 63105-000, Brazil; rayalmeidasilva2306@gmail.com (R.S.A.); priscilla.r.freitas@hotmail.com (P.R.F.); caroljustino@outlook.com (A.C.J.A.); Irwin.alencar@urca.br (I.R.A.M.); Eduardo.l.santos@kroton.com.br (E.L.S.); saulo.tintino@urca.br (S.R.T.); talysson97f.moura@gmail.com (T.F.M.); 2 Gonçalo Moniz Institute, Oswaldo Cruz Foundation (IGM-FIOCRUZ/BA), Salvador 40296-710, Brazil; jaime.ribeiro@fiocruz.br; 3 Department of Biomedicine, Christus University Center-Unichristus, Fortaleza 60190-060, Brazil; vitoriaferreira057@outlook.com; 4 Department of Biomedicine, Maurício de Nassau College-UNINASSAU, Petrolina 58401-115, Brazil; 011800513@prof.uninassau.edu.br; 5 Post Graduate Program in Sustainable Territorial Development, Federal University of Paraná–UFPR, Matinhos 59950-000, Brazil; luizeverson@ufpr.br (L.E.S.); wdoamaral@ufpr.br (W.d.A.); 6 Department of Chemistry, Post Graduate Program in Agronomy, Federal University of Paraná-UFPR, Curitiba 80011970, Brazil; cicero@ufpr.br; 7 Department of Agricultural and Environmental Sciences, Milan State University, via G. Celoria 2, 20133 Milan, Italy

**Keywords:** ethidium bromide, bacterial resistance, biological activity, chemical composition

## Abstract

Considering the evidence that essential oils, as well as safrole, could modulate bacterial growth in different resistant strains, this study aims to characterize the phytochemical profile and evaluate the antibacterial and antibiotic-modulating properties of the essential oil *Ocotea odorífera* (EOOO) and safrole against efflux pump (EP)-carrying strains. The EOOO was extracted by hydrodistillation, and the phytochemical analysis was performed by gas chromatography coupled to mass spectrometry (GC-MS). The antibacterial and antibiotic-modulating activities of the EOOO and safrole against resistant strains of *Staphylococcus aureus, Escherichia coli* and *Pseudomonas aeruginosa* were analyzed through the broth microdilution method. The EP-inhibiting potential of safrole in association with ethidium bromide or antibiotics was evaluated using the *S. aureus* 1199B and K2068 strains, which carry genes encoding efflux proteins associated with antibiotic resistance to norfloxacin and ciprofloxacin, respectively. A reduction in the MIC of ethidium bromide or antibiotics was used as a parameter of EP inhibition. The phytochemical analysis identified 16 different compounds in the EOOO including safrole as the principal constituent. While the EOOO and safrole exerted clinically relevant antibacterial effects against *S. aureus* only, they potentiated the antibacterial activity of norfloxacin against all strains evaluated by our study. The ethidium bromide and antibiotic assays using the strains of *S. aureus* SA1119B and K2068, as well as molecular docking analysis, indicated that safrole inhibits the NorA and MepA efflux pumps in *S. aureus.* In conclusion, *Ocotea odorifera* and safrole presented promising antibacterial and antibiotic-enhancing properties, which should be explored in the development of drugs to combat antibacterial resistance, especially in strains bearing genes encoding efflux proteins.

## 1. Introduction

Compounds generated by the secondary metabolism of plants constitute a large group of substances with significant structural and functional diversity, among which essential oils are notable bioactive compounds with antifungal, antiviral, antiprotozoal and antibacterial properties [1,2,3,4]. Essential oils are complex mixtures of volatile and aromatic compounds found in a great variety of plant species, acting in defense against infections, parasites, and other stress conditions [5,6]. In this context, terpenes, which comprise the largest class of natural products, have been identified as very potent bioactive compounds [7]. Thymol and carvacrol are notable compounds with potent antimicrobial activities. These monoterpenes have been found as major constituents of essential oils obtained from a wide variety of aromatic plants [8]. Accordingly, the essential oil of the leaves of Aloysia gratissima and Baccharis reticulata, which have 1,8-cineole, germacrene D, α-pinene, β-caryophyllene, and β -pinene as major constituents, demonstrated bactericidal activity against both Gram-positive (*Staphylococcus aureus* and *Bacillus cereus*) and Gram-negative (*Escherichia coli* and *Pseudomonas aeruginosa*) bacterial strains [9,10].

*Ocotea odorífera* (Lauraceae) is a plant popularly known as “sassafras”. This species, native to Brazil, is widely found in the Atlantic Forest where is used by the local communities in the treatment of malaria and rheumatism. In addition, due to its remarkable chemical constitution and abundance of essential oils, this plant has been used as a source of flavoring agents in the food industry [11,12,13]. Previous research with the essential oil of *O. odorifera* (EOOO) has identified safrole (C_10_H_10_O_2_) as a major constituent, with unique chemical and pharmacological properties [14,15,16]. Accordingly, safrole has been used in the production of fragrances and as a raw material in the synthesis of drugs and insecticides [17]. While the effects of essential oils containing safrole against Gram-negative strains of *Escherichia coli, Salmonella thyphimurium*, and *Pseudomonas aeruginosa*, have been demonstrated previously, the antibacterial activity of this organic compound against *Staphylococcus aureus* strains remains to be characterized [18,19,20].

*S. aureus* is a Gram-positive bacterium with remarkable pathogenicity. Accordingly, resistance to antibiotics is currently a major worldwide public health problem [21]. Resistant bacteria are characterized by the presence of natural or acquired mechanisms that confer survivability even in the presence of high concentrations of antibiotics [22]. In this context, genetic modification of the binding site, enzymatic inactivation, and active transport by efflux pumps (EPs) are recognized as the principal mechanisms of resistance to antibiotics [23]. Importantly, EPs were found to mediate antibiotic resistance in several strains of *S. aureus*. By actively transporting drugs to the extracellular medium, these transmembrane proteins reduce the intracellular concentrations of antibiotics, resulting in ineffective therapy [24].

Aiming to discover new weapons to combat bacterial resistance, researchers have been dedicated to investigating the antibacterial and antibiotic-modulating properties of natural products. Therefore, considering the evidence that essential oils and safrole could modulate bacterial growth in different resistant strains, this study aims to characterize the phytochemical profile and evaluate the antibacterial and antibiotic-modulating properties of the essential oil of *Ocotea odoriferas* and safrole in EP-carrying strains.

## 2. Results

### 2.1. Chemical Composition of the Essential Oil of Ocotea Odorífera

The extraction of the EOOO by hydrodistillation presented a yield of 2.31%, considering the dry weight of the botanical material. Phytochemical analysis of the essential oil through gas chromatography coupled with mass spectrometry (GC-MS) identified 93.1% of the total constituents, revealing the presence of 16 different compounds, including safrole (77.9%), spathulenol (4.0%) and ortho-cymene (3.0%) as major constituents (Table 1).

### 2.2. Antibacterial Activities of the EOOO and Safrole

The broth dilution method was used to determine the Minimum Inhibitory Concentration (MIC) of the essential oil of *Ocotea odorifera* and its major constituent safrole against multi-resistant strains of *S. aureus*, *E. coli* and *P. aeruginosa*. The antibacterial activity analysis revealed that both treatments presented MIC values above 1024 μg/mL against *E. coli* and *P. aeruginosa*, indicating that they exert clinically ineffective antibacterial activity against these strains. However, both treatments presented MIC values of 512 μg/mL against *S. aureus*, suggesting that *O. odorifera* and its major constituent safrole exert antibacterial effects against this Gram-positive strain (Table 2).

### 2.3. Antibiotic-Potentiating Effects of the EOOO and Safrole

**Figure 1:**
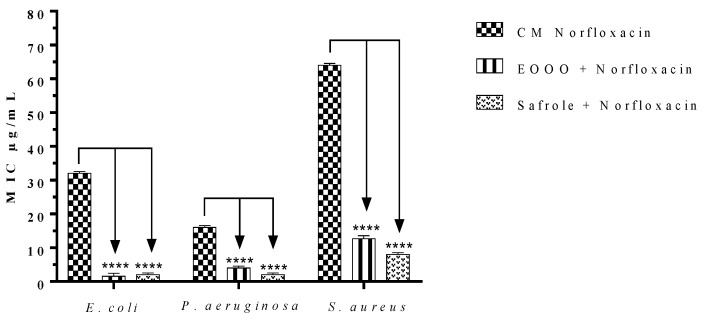
Minimum Inhibitory Concentration (MIC) of norfloxacin alone or in the presence of *O. odorífera* or safrole against the *multiresistant strains E. coli* 06, *S. aureus* 10 and *P. aeruginosa* 24. **** *p* < 0.0001 indicates significant differences between groups. Statistical significance was determined by one-way ANOVA and Bonferroni’s post-hoc test.

As the EOOO and safrole presented variable intrinsic antibacterial activity, this study analyzed the ability of these substances to modulate the antibacterial resistance to norfloxacin, a fluoroquinolone antibiotic. To this end, the MIC of this antibiotic was calculated after culturing the same bacterial strains in the presence or absence of the natural products at concentrations equivalent to their MIC ÷ 8. As shown in Figure 1, The MIC values of norfloxacin against strains of *S. aureus, P. aeruginosa* and *E. coli* were significantly reduced by both EOOO and safrole, indicating that they present antibiotic-modulating effects against all investigated strains. Interestingly, while these treatments did not present direct antibacterial effects against *P. aeruginosa* and *E. coli*, they were found to modulate the antibacterial resistance to norfloxacin observed for the Gram-positive and Gram-negative strains evaluated by the present study.

### 2.4. Effects of Safrole on the S. aureus NorA and MepA Efflux Proteins

**Figure 2:**
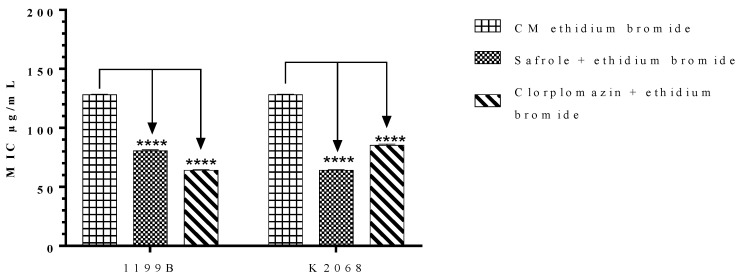
Minimum Inhibitory Concentration (MIC) of ethidium bromide alone or associated with safrole or chlorpromazine (control) against *S. aureus* 1199B and K2068 strains. **** *p* < 0.0001 indicates significant differences between groups. Statistical significance was determined by one-way ANOVA and Bonferroni’s post-hoc test.

The ethidium bromide assay has been widely used to evaluate the potential action of drugs as EP inhibitors [25]. Therefore, it was evaluated whether safrole could modulate bacterial resistance in the *S. aureus* 1199B and K2068 strains, which express the NorA and MepA EP, respectively. The association with subinhibitory concentrations of safrole or chlorpromazine (control drug) significantly reduced the MIC of ethidium bromide against both strains, indicating that safrole could act as an EP inhibitor in some *S. aureus* strains (Figure 2).

**Figure 3:**
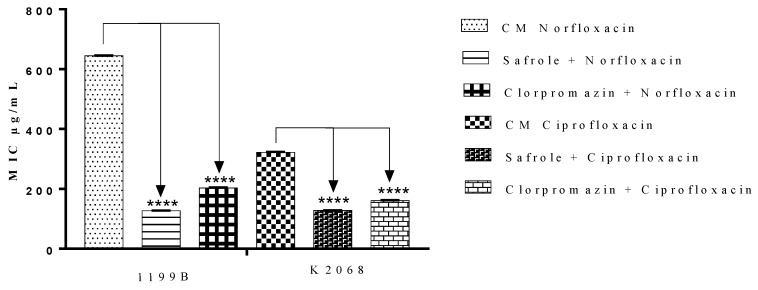
Minimum Inhibitory Concentration (MIC) by safrole in association with norfloxacin or ciprofloxacin against *S. aureus* 1199B and K2068 strains. **** *p* < 0.0001 indicates significant differences between groups. Statistical significance was determined by one-way ANOVA and Bonferroni’s post-hoc test.

Following the promising effects demonstrated by safrole in the ethidium bromide test, the effects of this compound as a modulator of bacterial resistance in association with norfloxacin and ciprofloxacin was investigated. Of note, the 1199B and K2068 strains, respectively, bear resistance genes against each of these antibiotics. To this end, the MIC of this antibiotic was calculated after culturing the same bacterial strains in the presence or absence of the natural products at concentrations equivalent to their MIC ÷ 8. As shown in Figure 3, the association with safrole or chlorpromazine (control) significantly reduced the MICs of both antibiotics, suggesting that the resistance to these drugs was, at least partially, reverted by safrole, which possibly inhibits the NorA and MepA-mediated efflux mechanisms in *S. aureus.*

### 2.5. Molecular Docking and Analysis of Knteractions between Safrole and Efflux Proteins

The docking simulations determined the ligand-bound protein complexes with minimal energy and the best stability. The best-docked ligand conformations were saved in output clusters 0. The docking studies revealed that chlorpromazine and safrole presented the most favorable interaction energies (IE), which were positively correlated with their MIC values, inhibition constants (Ki) and size-independent ligand efficiency (SILE) for both NorA (Table 3) and MepA (Table 4) efflux proteins. Together, this data shows that safrole has a more favorable interaction with the MepA protein compared to NorA.

**Figure 4:**
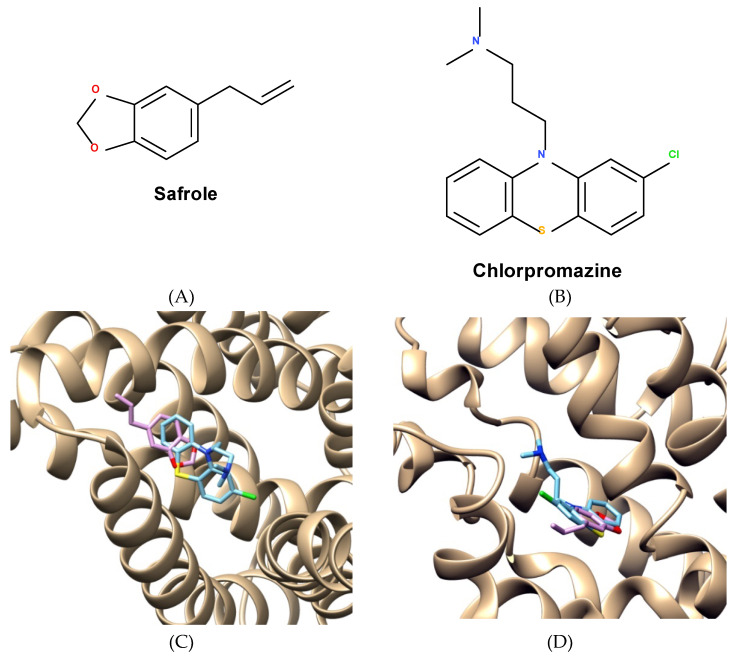

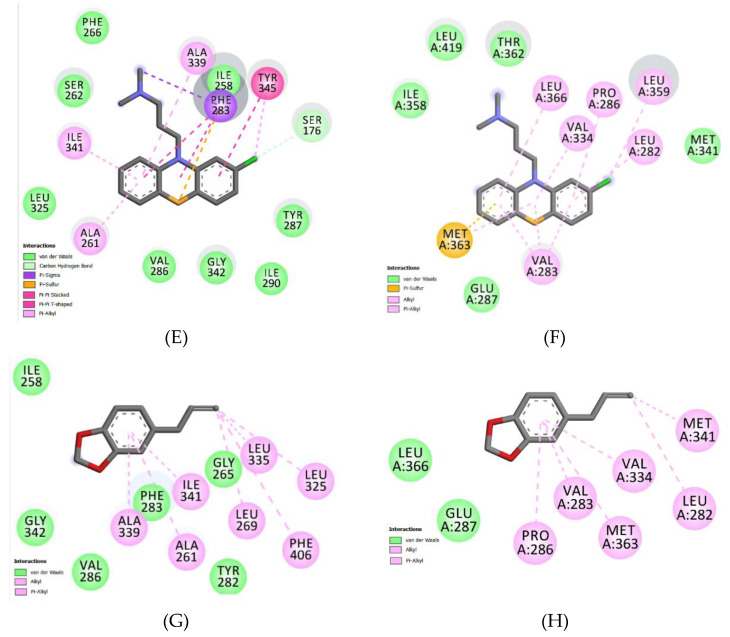
Chemical structures of Safrol (**A**) and Chlorpromazine (**B**). Binding poses of best stability of safrole and chlorpromazine with the NorA (**C**) and MepA (**D**). Interaction maps showing the binding of chlorpromazine to amino acid residues in the NorA (**E**) and MepA (**F**) binding sites. Interaction maps showing the binding of safrole to amino acid residues in the NorA (**G**) and MepA (**H**) binding sites.

Figure 4 shows the chemical structures of safrole (Figure 4A) and chlorpromazine (Figure 4B) in the binding pockets of the NorA (Figure 4C) and MepA (Figure 4D) efflux pumps. Interaction maps were used to predict the participation of amino acid residues in the binding of chlorpromazine (Figure 4E,F) or safrole (Figure 4G,H) to these efflux pumps. The interaction maps show that chlorpromazine and safrole similarly interact with the MepA binding site through anchors with Glu287, Leu366, Met363, Met341 Pro286, Val283, Leu282, and Val334, as well as with the NorA binding site through Gly342, Val286, Ile258, Ala261, Ile341, Ala339, Leu325, and Phe283. The interactions between these amino acids and safrole are given at nonpolar and partially hydrophobic regions involving Van der Waals, π-Allyl, and Allyl interactions. On the other hand, the binding of chlorpromazine to the corresponding amino acids predominantly involves Van der Waals, π-Allyl, Allyl, carbon hydrogen bond, π-π, π-sigma, and π-sulfur interactions. These findings corroborate the evidence that safrole, as well as chlorpromazine, could act as efflux pump inhibitors in the *S. aureus* SA1119B and K2068 strains.

## 3. Discussion

The discovery of penicillin represented an important milestone in the therapy of bacterial diseases and opened new perspectives for antibacterial drug development. Additionally, the introduction of the last generation of antibiotics has had a significant impact on public health, contributing to reduced morbidity and mortality rates caused by bacterial infections. However, the irrational use of this therapeutic classes has contributed to the selection of multidrug-resistant bacterial strains, against which conventional antibiotics may become ineffective. Therefore, the discovery of new antibiotics is crucially important to ensure the success of antibiotic therapy in future medicine [26,27,28].

The present research investigated the antibacterial effects of the essential oil of *O. odorifera* and its major constituent safrole against multi-resistant strains. The phytochemical analysis identified 93.1% of the total constituents of the EOOO, revealing the presence of 16 different compounds, including safrole as a major constituent. This finding is corroborated by a previous study showing a similar composition, as well as the presence of safrole as the principal component of essential oil obtained from the leaves of the same species [29]. According to Junior et al. [30] and Lorenzi and Matos [31], safrole is a bioactive compound widely used in the food, cosmetic, and pharmaceutical industries. In general, essential oils present significant yield, and their composition can vary depending on climatic and environmental factors, such as temperature, humidity, precipitation, soil, and time of collection. Additionally, the part of the plant used (leaves, bark, seeds or root), as well as the method of the extraction, may interfere with the composition of the essential oil of a given species [15,32,33].

The antibacterial activity analysis revealed that while the essential oil of *O. odorifera* and safrole presented clinically ineffective antibacterial activity against *E. coli* and *P. aeruginosa*, they presented clinically relevant MIC values against *S. aureus*. The data obtained by this study suggest that safrole is, at least partially, responsible for the antibacterial effects of the EOOO. It is still suggested that both the oil and the isolated compound are more effective against Gram-positive strains, which may be justified by differences in the constitution of the membrane of Gram-positive and Gram-negative bacteria.

According to Nazzaro et al. [34], Gram-negative bacteria are more resistant to the penetration of essential oils due to the presence of an outer layer of lipopolysaccharides. Accordingly, Betim et al. [35], comparing the antibacterial activities of *O. odorífera* and *O. nutans*, demonstrated that the essential oil of *O. odorífera* presented more potent effects. In addition, they found that both essential oils were more effective against Gram-positive bacteria, corroborating the findings of the present research. On the other hand, a study by Damascemo et al. [36] found clinically ineffective MIC values for the essential oil of *O. bicolor* against *S. aureus, P. aeruginosa*, and *E. coli*. Furthermore, Cansian et al. [37] found that an essential oil obtained from *O. odorifera* was more efficient against Gram-negative bacteria. As discussed above, the chemical composition of a species may be significantly influenced by several environmental factors, which may affect the pharmacological effects of extracts, fractions, and essential oils.

Following the antibacterial activity analysis, this work investigated the ability of the EOOO and safrole ability to modulate the antibacterial resistance to norfloxacin, a fluoroquinolone antibiotic. The MIC values of norfloxacin against strains of *S. aureus, P. aeruginosa*, and *E. coli* were significantly reduced in the presence of a subinhibitory concentration of the EOOO and safrole (MIC ÷ 8) indicating that both the oil and the isolated compound can modulate the antibacterial resistance to norfloxacin observed for the Gram-positive and Gram-negative strains evaluated by our study. Nevertheless, the isolated compound showed more potent antibiotic-enhancing activity against all strains, reducing the MIC of norfloxacin by up to 7 fold in comparison with the antibiotic alone. Studies have demonstrated that antibacterial resistance to norfloxacin is significantly mediated by the expression of efflux systems [38]. Therefore, it is hypothesized that the antibiotic-enhancing effects shown by the EOOO and safrole might involve inhibition of efflux pumps.

Efflux pumps are membrane proteins that carry out the active transport of a wide range of molecules, removing potentially toxic substances from the intracellular medium. However, in the context of antibiotic therapy, the active transport of drugs by these proteins results in reduced intracellular concentrations and, consequently, failure in the therapeutic effect [23]. Increasing evidence has demonstrated that efflux pumps are overexpressed in multiresistant bacterial strains, such as methicillin-resistant *Staphylococcus aureus* (MRSA). Thus, considering the notable role of efflux pumps on antibacterial resistance in *S. aureus,* we evaluated the potential involvement of EP inhibition on safrole-mediated antibiotic resistance modulation in the *S. aureus* 1199B and K2068 strains, which express the NorA and MepA EP, respectively. In the present study, the association with subinhibitory concentrations of safrole or chlorpromazine (control drug) significantly reduced the MIC of ethidium bromide against both strains. According to Tintino et al. [39], when natural or synthetic compounds are tested against EP-bearing strains in association with ethidium bromide, a reduction in the MIC of this substance indicates that the tested compound also acts as an EP inhibitor. Thus, it is suggested that safrole could serve as an EP inhibitor in these *S. aureus* strains.

Accordingly, Oliveira-Tintino et al. [40] reported that while the essential oil of *Chenopodium ambrosioides* significantly reduced the MIC of ethidium bromide against the 1199B strain, its principal constituent α-Terpinene presented no significant modulating effect, suggesting that other components of the *C. ambrosioides* oil could act as EP inhibitors. However, the molecular mechanisms associated with EP inhibition by essential oils, as well as their isolated components, remain to be fully characterized. Nevertheless, since ethidium bromide is used as a substrate by bacterial EP, it is hypothesized that these natural products could act by blocking the binding of the substrate to the pump. Additionally, it has been suggested that energy depletion, competition with ATP, and interference with the proton gradient are potential mechanisms associated with EP inhibition by natural or synthetic compounds [41].

Considering the promising effects demonstrated by safrole in the efflux pump inhibition assay, it was evaluated whether safrole could modulate bacterial resistance *S. aureus* strains bearing resistance genes against norfloxacin and ciprofloxacin. The results of the present study demonstrated that safrole, as well as the EP inhibitor chlorpromazine (control), significantly reduced the MICs of both antibiotics, suggesting that the resistance to these drugs was, at least partially, reverted by safrole, which possibly inhibits the NorA- and MepA-mediated efflux mechanisms in *S. aureus* [42,43]. To confirm this hypothesis, molecular modeling and docking analysis were used to evaluate in silico, the potential interactions between safrole or chlorpromazine (control drug) and the NorA and MepA efflux pumps. The present findings suggest that safrole, as well as chlorpromazine, could act as efflux pump inhibitors in the *S. aureus* SA1119B and K2068 strains, and that safrole has a more favorable interaction with the MepA protein compared to NorA.

Previous studies suggest that chlorpromazine affects the membrane potassium flow in *S. aureus*. However, in multi-drug resistant strains, a significant inhibition is observed only at concentrations above 50 µM [44]. Chlorpromazine is a typical antipsychotic drug acting as a dopamine antagonist. Curiously, studies have shown that this drug also acts as a strong inhibitor of the NorA efflux pump, and there is evidence that the interaction between this drug and the amino acid residues is favored by its hydrophobic properties and molecular dimension, corroborating the findings shown in the interaction map [45].

Finally, structure-activity relationship (SAR) studies indicate that phenyl-ether groups significantly contribute to the interactions with the NorA and MepA EP in *S. aureus* [46]. Additionally, accumulating evidence suggest that high lipophilicity facilitates the action of EP inhibitors on these proteins, corroborating the findings of the present research [43].

## 4. Materials and Methods

### 4.1. Collection and Identification of the Botanic Material

**Figure 5:**
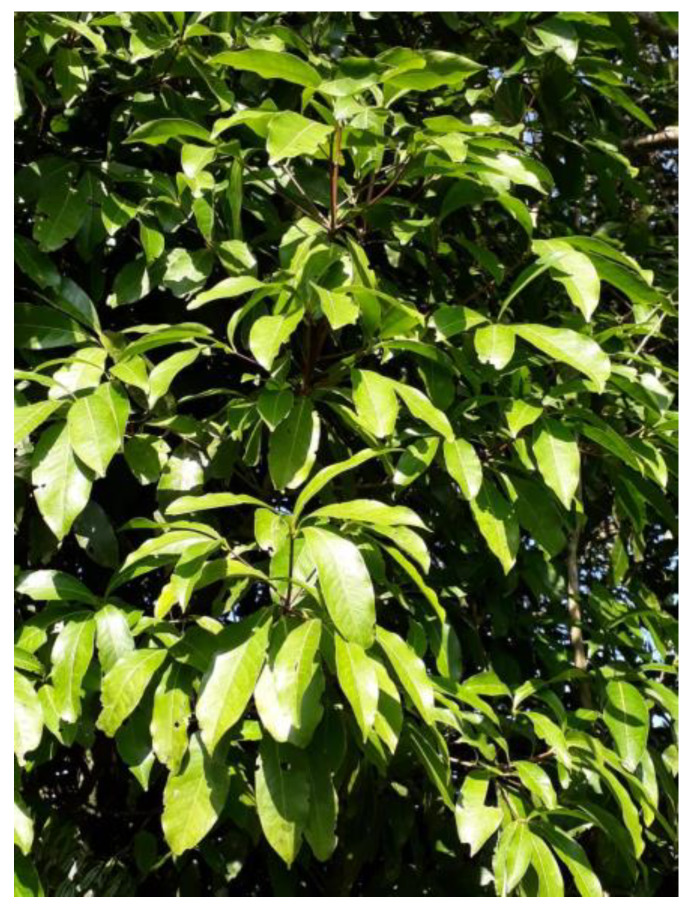
Sample of the plant *Ocotea odorífera.*

The essential oil was extracted from terminal branches and inflorescences of plants (Figure 5) collected in a segment of Atlantic Forest in the State of Paraná, Southern Brazil, located at the following geographical coordinates: S 25° 19.862′ W 49° 48.338′. The collection and transport of the plant samples were performed under the authorization of the Paraná Environmental Institute (registry number 284/11). A voucher specimen was prepared and registered at the Herbarium of “Faculdades Integradas Espirita” (HFIE) (registry number 9.000). Terminal branches and inflorescences were randomly collected from at least 10 individual plants and dried with an electric dryer (Gama Italy IQ perfetto 127V) at 40 °C for 24 h.

### 4.2. Essential Oil Extraction

The essential oil of *O. odoriferous* was extracted by hydrodistillation in a Clevenger type apparatus. Briefly, approximately 1 kg of the plant material was crushed and subjected to extraction with 2.5 L of distilled water at boiling temperature for 2 h. After extraction, the essential oil was combined with anhydrous sodium sulfate (Na_2_SO_4_) and stored under refrigeration (−4 °C) for preservation [47].

### 4.3. Calculation of Essential Oil Yield

The yield of the essential oil was calculated as a percentage of the dry biomass obtained from the aerial parts of the plant, using the following equation:

(1) $$TO=\frac{VO}{MS}\;\times \;100$$

Legend: *TO* = oil content in 100 g of biomass; *VO* = volume of oil obtained; *MS* = amount of dry biomass, free of water and humidity; and 100 = conversion factor to percentage [48].

### 4.4. Phytochemical Analysis

The chemical composition of the essential oil was determined by gas chromatography coupled to mass spectrometry (CG-EM) using an Agilent 6890 chromatograph (Palo Alto, CA, USA) coupled to an Agilent 5973N mass selection detector. The separation of the constituents was obtained in a capillary column HP-5MS (5%-phenyl-95%-dimethylpolysiloxane, 30 m × 0.25 mm × 0.25 μm) and using helium as the carrier gas (1.0 mL min^−1^). The chemical constituents were identified by comparing their mass spectra with the standards reported in the literature [49].

### 4.5. Bacterial Cultures

The following multidrug-resistant strains were used in the antibacterial tests: *Pseudomonas aeruginosa* 24, *Staphylococcus aureus* 10 and *Escherichia coli* 06. The origin and resistance profile of these strains is shown in Table 5.

*S. aureus* strains 1199B and K2068, which carry the NorA and MepA efflux proteins, respectively, were kindly provided by Prof. S. Gibbons (University of London). All strains were initially kept on blood agar (Laboratorios Difco Ltd.a., São Paulo, Brazil) and maintained in Heart Infusion Agar (HIA, Difco) medium at 4 °C. Samples were transferred from the solid medium to test tubes containing sterile saline, and turbidity was assessed using a value of 0.5 on the McFarland scale, corresponding to 10^5^ CFU.

### 4.6. Drugs

Norfloxacin and ciprofloxacin were used in the tests with the 1199B and K2068 strains, respectively. These strains carry the NorA and MepA efflux proteins, respectively, which confer resistance to the corresponding antibiotic. Ethidium bromide was used as an efflux pump inhibitor control. Both antibiotics were dissolved in DMSO and diluted in water, while ethidium bromide was dissolved in water. All drugs were prepared at initial concentrations of 1024 µg/mL and serially diluted in test tubes. Both antibiotics, ethidium bromide and safrole were purchased from SIGMA Chemical Co. (St. Louis, MO, USA).

### 4.7. Determination of Minimum Inhibitory Concentration (MIC)

Each inoculum was prepared with 10% Brain Heart Infusion (BHI) at a ratio of 1:9. Next, 100 µL of inoculum in medium was placed in wells on a 96-well plate with 100 µL of the substance at concentrations ranging from 1024 to 8 µg/mL, followed by incubation at 37 °C for 24 h. Positive controls (medium + inoculum) were included in the last wells of the plate [50]. After incubation, 20 µL of sodium resazurin was added to each well, followed by an additional 1 h incubation period at room temperature. A change in the color of the solution, due to the reduction of resazurin, was used as an indicator of bacterial growth [51,52]. The MIC was defined as the lowest concentration capable of inhibiting bacterial growth. All experiments were carried out in triplicate for all bacterial strains.

### 4.8. Analysis of Antibiotic Resistance Modulation

To evaluate the ability of the EOOO and safrole to modulate bacterial resistance in the presence of other drugs, the MICs of norfloxacin and ciprofloxacin against resistant strains of *P. aeruginosa*, *E. coli*, and *S. aureus* were determined in the presence or absence of these natural products at concentrations equivalent to their MIC ÷ 8 [53]. The readings were performed as described above.

### 4.9. Efflux Pump Inhibition Analysis Using an Ethidium Bromide Assay

In this test, the MIC of ethidium bromide was determined in the presence or absence of sub-inhibitory concentrations of the EOOO and safrole. Briefly, the bacterial inocula were prepared in BHI medium, and the treatments were added at concentrations equivalent to their MIC ÷ 8. Wells on a 96-well plate were filled with 100 µL of the solutions of each treatment and then, ethidium bromide was added to the wells at concentrations ranging from 1024 to 0.5 µg/mL. A reduction in the ethidium bromide MIC was interpreted as EP inhibition [54]. Experimental controls and MIC values for both OEOC and safrole were determined as described above. All tests were performed in triplicate.

### 4.10. Efflux Pump Inhibition Analysis Using an Antibiotic Resistance Modulation Assay

Considering that the *S. aureus* strains 1199B and K2068 carry genes encoding efflux proteins associated with antibiotic resistance to norfloxacin and ciprofloxacin, respectively, this protocol was used to evaluate the ability of the EOOO and safrole to modulate bacterial resistance in association with these antibiotics against the corresponding strain. The bacterial inocula were prepared in BHI medium and the treatments were added at concentrations equivalent to their MIC ÷ 8. Wells on a 96-well plate were filled with 100 µL of the solutions of each treatment and then, each antibiotic was added to the wells at concentrations ranging from 1024 to 0.5 µg/mL. A reduction in the MIC of the antibiotic was interpreted as EP inhibition [54]. Experimental controls and MIC values for both EOOO and safrole were determined as described above. All tests were performed in triplicate.

### 4.11. Molecular Modelling and Docking Studies

The three-dimensional (3D) structures of the NoRA and MepA proteins were determined using homology models as follows: the three-dimensional structures were predicted using the Protein Homology/Analogy Recognition Engine version 2.0 (Phyre2, available http://www.sbg.bio.ic.ac.uk/phyre2) web server, and confirmed using the Iterative Threading Assembly Refinement (I-TASSER, available http://zhanglab.ccmb.med.umich.edu/I-TASSER/) and MODELLER 9.14 servers. The stereochemical quality of the protein structures was checked by the Ramachandran plot using the PROCHECK program [55]. The topographic structure of the pockets and cavities were characterized using the Computed Atlas of Surface Topography of proteins (CASTp, available http://cast.engr.uic.edu) with a standard value of 1.4 Å of the radius, and the ligand-binding sites were predicted using the 3DligandSite server [56].

Docking simulations were carried out with a binding region defined by a 10 Å × 10 Å × 10 Å box set at the centroid of predicted ligand-binding sites. The structures were adjusted using a protein preparation tool provided by the Chimera package (version 1.14, University of California, San Francisco, CA, USA), the 3D structures of ligands were obtained using the corina^®^ 3D structure generator (version 4.3, Altamira LLC, Oklahoma city, OK, USA), and minimization of energy was achieved using the UCSF Chimera structure build module [57]. The docking analyses were carried out using the UCSF Chimera and AutoDock Vina software (version 1.1.2., The Scripps Research Institute, La Jolla, CA, USA) based on the iterated local search global optimizer [58]. Proteins and ligands were maintained flexible during the docking process. The selection of flexible residues from proteins was based on the active site at 4.0 Å from the co-crystallized ligands. The most favorable binding free energy was represented by clustering the positional Root-mean-square deviation of atomic positions (RMSD) data with no more than 1.0 Å. The final docked complexes were analyzed using the Discovery Studio visualizer program version 3.1 (Dassault Systèmes, San Diego, CA, USA). The binding energy score was used to calculate the inhibition constant (Ki value)-based in an equation by Onawole et al. [59], and the size-independent ligand efficiency (SILE) was measured using the equation described by Nissink, J. W. M. [60].

### 4.12. Statistical Analysis

Data are expressed as arithmetic means ± standard error of the mean and were analyzed by analysis of variance (ANOVA), followed by Bonferroni’s post-test using GraphPad Prism software version 7.00 (available http://www.graphpad.com/scientific-software/prism/). Statistical significance was considered when *p* < 0.05.

## 5. Conclusions

The essential oil of *Ocotea odorifera* has antibacterial and antibiotic-enhancing activities that are, at least partially, mediated by its major constituent, safrole. Safrole modulates antibiotic resistance in the *S. aureus* SA1119B and K2068 strains, possibly through direct inhibition of the NorA and MepA efflux pumps, respectively.

In conclusion, *Ocotea odorifera* and safrole present promising antibacterial and antibiotic-enhancing properties, which should be explored in the development of drugs to combat antibacterial resistance, especially in strains bearing genes encoding efflux proteins.

## Figures and Tables

**Table 1:** GC-MS profile of the essential oil of *Ocotea odorífera*.

RI	Compound	%
936	Alpha-pinene	0.3
951	Camphene	0.2
978	Beta-pinene	0.1
1005	Alpha-felandrene	1.9
1026	Ortho-cymene	3.0
1033	1,8-Cineole	0.9
1145	Camphor	0.4
1189	Alfa-terpineol	0.3
1292	Safrole	77.9
1356	Eugenol	0.6
1414	(*E*)-caryophyllene	0.4
1476	Gama-muurolene	0.3
1487	Delta-selinene	0.5
1491	Bicyclogemacrene	1.1
1572	Spathulenol	4.0
1648	11-selinen-4-alpha-ol	1.2
**Total composition identified**		**93.1**

Legend: RI = Retention Index.

**Table 2:** Minimum Inhibitory Concentrations (MICs) of the EOOO and safrole.

Bacterial Strain	EOOO MIC(μg/mL)	SafroleMIC (μg/mL)
*S. aureus* 10	512	512
*E. coli* 06	≥1024	≥1024
*P. aeruginosa* 24	≥1024	≥1024

**Table 3:** Molecular docking and analysis of interactions between EP inhibitors and NorA.

Compound	MIC (µg/mL)	IE (Kcal/mol)	Ki (µM)	SILE
Ethidium Bromide	128	−7.8	1.95	0.74
Chlorpromazine	64	−6.4	20.64	7.86
Safrole	80.63	−5.9	47.95	18.72

Legends: MIC = minimum inhibitory concentration; IE = interaction energy; Ki = inhibition constant; SILE = size independent ligand efficiency.

**Table 4:** Molecular docking and analysis of interactions between EP inhibitors and MepA.

Compound	MIC (µg/mL)	IE (Kcal/mol)	Ki (µM)	SILE
Ethidium Bromide	128	−8.6	0.51	0.19
Chlorpromazine	85.33	−6.9	8.88	3.47
Safrole	64	−6.1	34.23	15.19

Legends: MIC = minimum inhibitory concentration; IE = interaction energy; Ki = inhibition constant; SILE = size independent ligand efficiency.

**Table 5:** Origin and antibiotic resistance profile of the strains.

Bacterial Strain	Origin	Resistance Profile
*S. aureus* 10	Rectum swab	Amc, Amox, Amp, Asb, Azi, Cefa Cef, Cf, Cip, Cla, Clin, Ery, Lev, Mox, Oxa, Pen
*E. coli* 06	Urine	Asb, Cefa, Cef, Cfo, Cpm, Ctx
*P. aeruginosa* 24	Nasal discharge	Ami, Cip, Ctz, Imi, Lev, Mer, Ptz

Legend: Amc—Amoxicillin + Clavulanic Acid, Ami—Amikacin, Amox—Amoxicillin, Amp Ampicillin, Asb—Ampicillin + Sulbactam, Azi—Azithromycin, Cefa—Cefadroxil; Cef—Cephalexin, Cfo—Cefoxitin, Cip—Ciprofloxacin, Cla—Clarithromycin, Clin—Clindamycin, Cpm—Cefepime, Ctx—Ceftriaxone, Ctz—Ceftazidime, Ery—Erythromycin, Imi—Imipenem, Lev—Levofloxacin, Mer—Meropenem, Mox—Moxifloxacin, Oxa—Oxacillin, Pen—Penicillin and Ptz—Piperacillin.
